# Update from the 5th Edition of the World Health Organization Classification of Head and Neck Tumours: Tumours of the Ear

**DOI:** 10.1007/s12105-022-01450-9

**Published:** 2022-04-09

**Authors:** Ann Sandison

**Affiliations:** Guy’s & St Thomas’ NHS Foundation Trust, King’s College London, London, UK

**Keywords:** External ear and auditory canal, Middle ear, Inner ear, Ceruminous gland adenoma, Osteoma, Middle ear neuroendocrine tumour, Otosclerosis, Cholesteatoma, Acoustic neuroma, Endolymphatic sac tumour

## Abstract

In the recently published 5th Edition of the World Health Organisation Classification of Head and Neck Tumours, there are relatively few changes to report in terms of nomenclature in lesions of ear and temporal bone and fewer developments in molecular pathogenesis in comparison to other sites, particularly in sinonasal tract. Ear and temporal bone tumours are rare and biopsy material is limited. As a result, resources in the literature are scarce with few large series, no controlled clinical trials and the approaches to staging and management are not standardised. New entities are difficult to characterise. The number of entries has, however, increased for tumours of the ear and temporal bone (thirteen) compared to the 4th Edition (eleven). Some lesions previously included in the 4th Edition considered to have no site-specific features have been excluded to be discussed elsewhere and other benign lesions that are specific to this site have been included. The tumours and tumour-like entities of ear and temporal bone are discussed here mindful that the chapter in the 5th edition better correlates disease processes with clinical information and imaging and as far as possible standardises nomenclature.

## Introduction

Tumours of the ear are traditionally discussed according to anatomical site in external ear, middle or inner ear as there are specific clinical and anatomical considerations for each site. This tradition is maintained in the new edition of World Health Organisation Classification of Head and Neck Tumours, however, lesions occurring in the middle and inner ear are discussed together, reflecting the clinical and radiological overlap and the frequent difficulty in determining the primary site.

In the external ear, site specific features of squamous cell carcinoma are discussed as well as the ceruminous gland tumours that are unique to the site. Cystic chondromalacia and chondrodermatitis nodularis chronica helicis have been included in this edition as benign tumoral lesions of the auricular cartilage and exostosis or osteoma of the external ear canal is discussed.

Middle ear papilloma is included as a separate entity and middle ear adenoma is renamed as middle ear neuroendocrine tumour (MeNet) to align the nomenclature with neuroendocrine tumours at other body sites.

A universally accepted staging system for carcinoma of the ear and temporal bone does not yet exist. The use of an evidence based minimum dataset and proposed pathological staging system for the reporting of tumours of ear and temporal bone, produced on behalf of the International Collaboration of Cancer Reporting (ICCR), is encouraged [1].

The entities included in the new edition are listed below highlighting key histological features and advances in the understanding of the anatomical considerations and pathogenesis of these uncommon lesions.

## Tumours of External Ear

### Chondrodermatitis Nodularis Chronica Helicis

Chondrodermatitis nodularis chronica helicis, historically known as Winkler's disease, is a benign painful condition affecting the pinna. Macroscopically a small nodule forms on the auricle, usually in the superior portion of the helix that may become ulcerated. Clinically and histologically the ulcerated nodule may be mistaken for squamous cell carcinoma. The disease is more common in males, middle aged or older but it can be seen in women and younger adults. The pathogenesis of the lesions is uncertain is probably related to cartilage ischaemia. Considering the helix is one of the furthest points from the source of the arterial blood supply of the pinna, it seems likely that obstruction of small arteries of the perichondrium is the primary lesion leading to cartilage necrosis with acute inflammation and epidermal ulceration secondary to that. An association between chondrodermatitis nodularis helicis and systemic sclerosis has been described. In this condition obstructive changes are frequently found in small arteries.

Development of the lesions has been attributed to sleeping on the affected side, and more recently using tight headgear or headphones and Bluetooth ear devices. It can occur after exposure to cold or after minor trauma [2].

Histological examination of representative biopsies, in which the elastic cartilage underlying the skin of the auricle is particularly well-represented, shows ulceration of the skin of the auricle and complete necrosis of the tip of the underlying elastic cartilage. In some cases, a piece of extruded necrotic cartilage may be seen on the floor of the ulcer. The perichondrium of the elastic cartilage in the region of the ulcer shows obstructive thickening of small arteries. Epithelium adjacent to the ulcer may show atypia and pseudoepitheliomatous hyperplasia and the main differential is squamous cell carcinoma. Treatment is surgical and the cosmetic implications are an important consideration.

### Cystic Chondromalacia

This benign cystic degenerative inflammatory condition affecting the auricle may be mistaken for chondrodermatitis nodularis chronica helicis and for squamous cell carcinoma. Lesions may be extensive, up to 4 cm and affect both ears usually not synchronously and may recur. Diagnosis depends on a representative biopsy of auricular cartilage. The main differential is chondrodermatitis nodularis chronica helicis.

### Exostosis (Osteoma)

Osteoma or exostosis are terms used interchangeably and refer to a benign bony enlargement of the deeper bony portion of the external auditory meatus. Biopsy material frequently does not allow the subtle difference between the lesions to be appreciated.

The lesions tend to develop in people who swim frequently thus exposing the tympanic bone to cold water. Exostosis is a more common broad-based lesion, which is often bilateral and symmetrical. Multiple osteomas may be seen in association with Gardner’s syndrome.

**Fig. 1:**
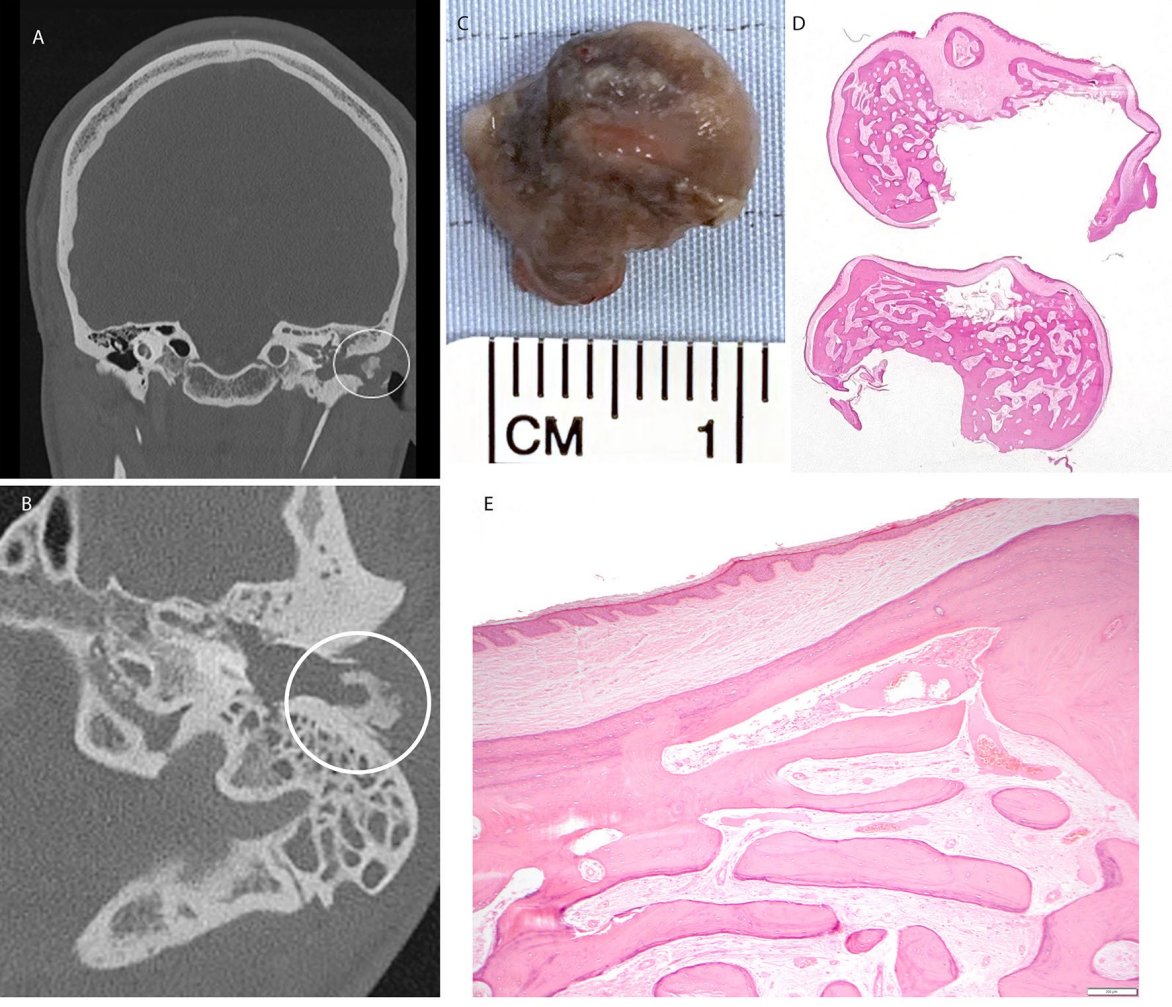
Osteoma. **A** Coronal image from CT showing osteoma of left ear canal (circled). **B** Axial CT image through the left temporal bone demonstrating an exophytic osseous mass arising from the dorsolateral bony external auditory meatus (circled) in keeping with an osteoma. **C** Macroscopic image of the resected lesion. **D** Wholemount, **E** low power H&E stained sections showing bland squamous epithelium overlying lamellar bone present as sclerotic cortical bone and anastomosing trabeculae of bone with intervening fibrovascular tissue

Exostosis is usually situated deeper in the ear canal than osteoma. The osteoma is composed of lamellar bone and may show outer cortical and inner cancellous trabeculated areas, the latter with marrow spaces (Fig. 1). There may be appositional new bone formation i.e., a thin layer of woven bone on the surfaces of the lamellar bone. The osteoma is covered by the normal squamous epithelium of the ear canal. Unlike osteoma the bone formations of exostosis do not possess marrow spaces.

### Ceruminous Gland Adenoma

**Fig. 2:**
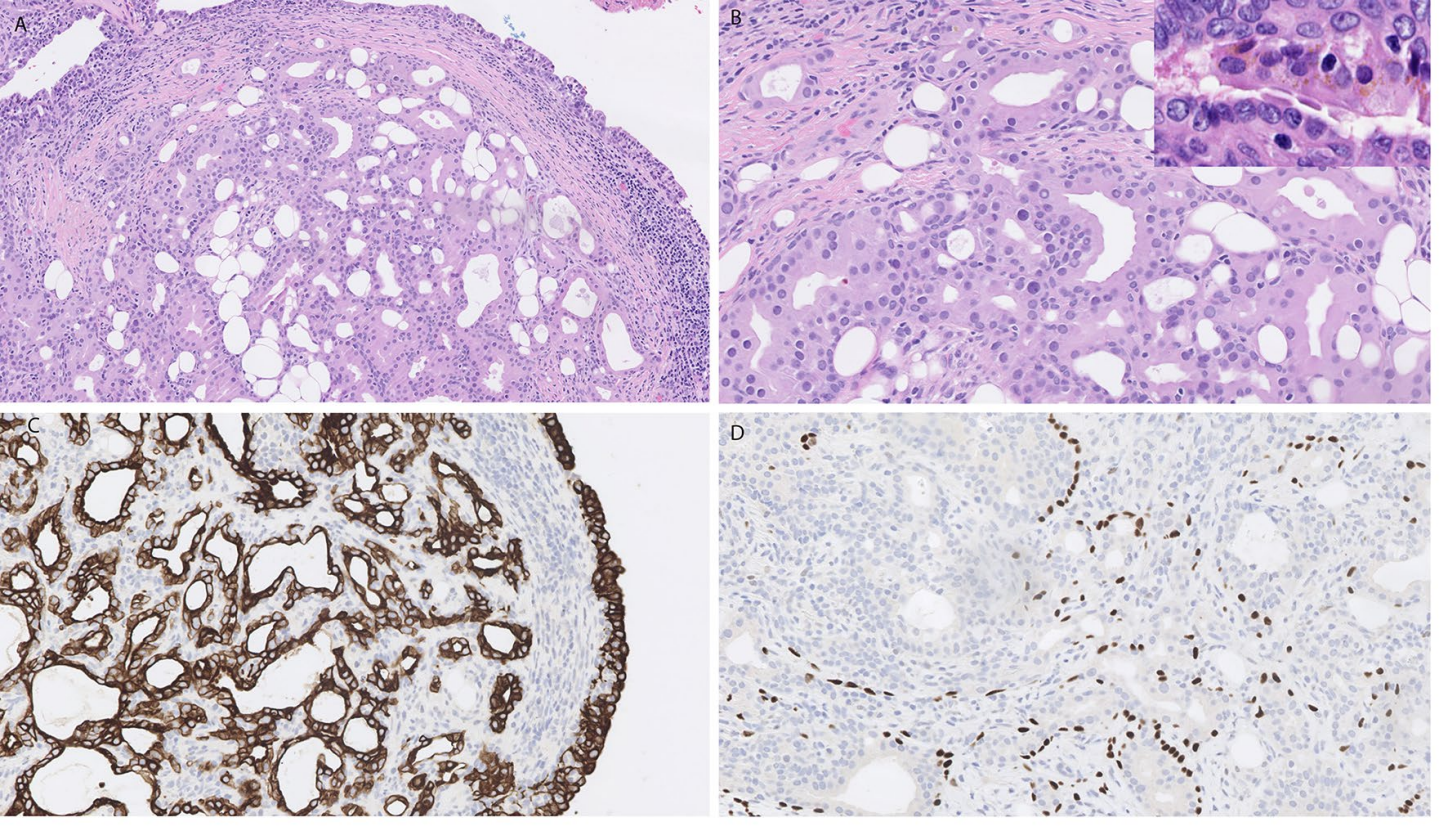
Ceruminous adenoma. **A**, **B** H&E stained sections showing a well circumscribed tumour in aural mucosa composed of focally back to back glands with luminal eosinophilic cells showing mild nuclear pleomorphism. Amber coloured ceroid pigment is present in some cells (inset **B**). **C** Immunostain for CK7 highlights the luminal cells within glands as well as surface epithelium. **D** p63 immunostain highlights a focal dual population of basal cells present around glands

Benign tumours of ceruminous glands are histologically categorised in the 5th edition as adenoma NOS, pleomorphic adenoma and syringocystoma papilliferum. The latter are histologically similar to the named tumour at other sites with additional features of ceruminous differentiation including presence of yellow ceroid pigment and decapitation secretion. The tumours are predominantly glandular, often with cystic change. Lesional cells extend into surrounding stroma, associated with inter-glandular fibrosis. Architecture may be solid, including back-to-back glands, and papillary. A dual cell population (inner/luminal epithelial cells and outer basal/myoepithelial cells) is sometimes visible on haematoxylin and eosin-stained sections and immunostains (Fig. 2). Tumours show low or moderate cellularity and mild nuclear pleomorphism. Nuclei are round to oval with fine, granular chromatin and small nucleoli. Luminal cells are columnar to cuboidal, with well-defined cell borders and abundant eosinophilic cytoplasm, usually showing apical caps and decapitation secretion. Most tumours contain cells with cytoplasmic golden-yellow/brown (ceroid) pigment granules.

Immunostains for pan cytokeratin are diffusely positive and luminal cells express CK7, epithelial membrane antigen (EMA), and CD117 and GATA 3. Myoepithelial/basal cells express p40, p63, CK5/6, S100 protein, SOX10 and SMA [3–5].

The main differentials are the malignant counterpart ceruminous adenocarcinoma and extension of middle neuroendocrine tumour into external ear.

### Ceruminous Gland Adenocarcinoma

This category includes malignant tumours arising from ceruminous glands of the external ear. Three distinct histological types are included, adenocarcinoma NOS, adenoid cystic carcinoma and mucoepidermoid carcinoma. Both adenoid cystic and mucoepidermoid carcinoma are histologically identical to the salivary counterparts.

Adenoid cystic carcinoma has been described confined to the middle ear and mastoid [6]. The tumours with predominantly tubular and cribriform morphology are low grade while those tumours with 30% or more of the tumour area represented showing solid morphology are considered to be high grade. Prognosis is not only dependent on morphology. The treatment is surgical and low-grade tumours occurring at inaccessible sites such as temporal bone may not be amenable to curative treatment. Fusions between MYB, MYBL1 and NF1B genes are associated with adenoid cystic carcinoma and are preserved in metastases [7]. The MYB-NF1B gene fusion upregulates MYB and appears to be specific to adenoid cystic carcinoma. Overexpression of MYB as detected on immunohistochemistry has been reported with variable specificity [8]. Positive immunostaining for MYB has been reported in several other tumours including basaloid squamous cell carcinoma, basal cell adenocarcinoma and epithelial myoepithelial carcinoma [9] as well as HPV associated sinonasal carcinoma [10]. In a recent study it has been proposed that negative immunostaining for MYB may identify tumours with poorer prognosis. The same study proposes that an increased risk of tumour associated mortality may be associated with cytoplasmic positivity for beta-catenin [11]^.^

### Squamous Cell Carcinoma

**Fig. 3:**
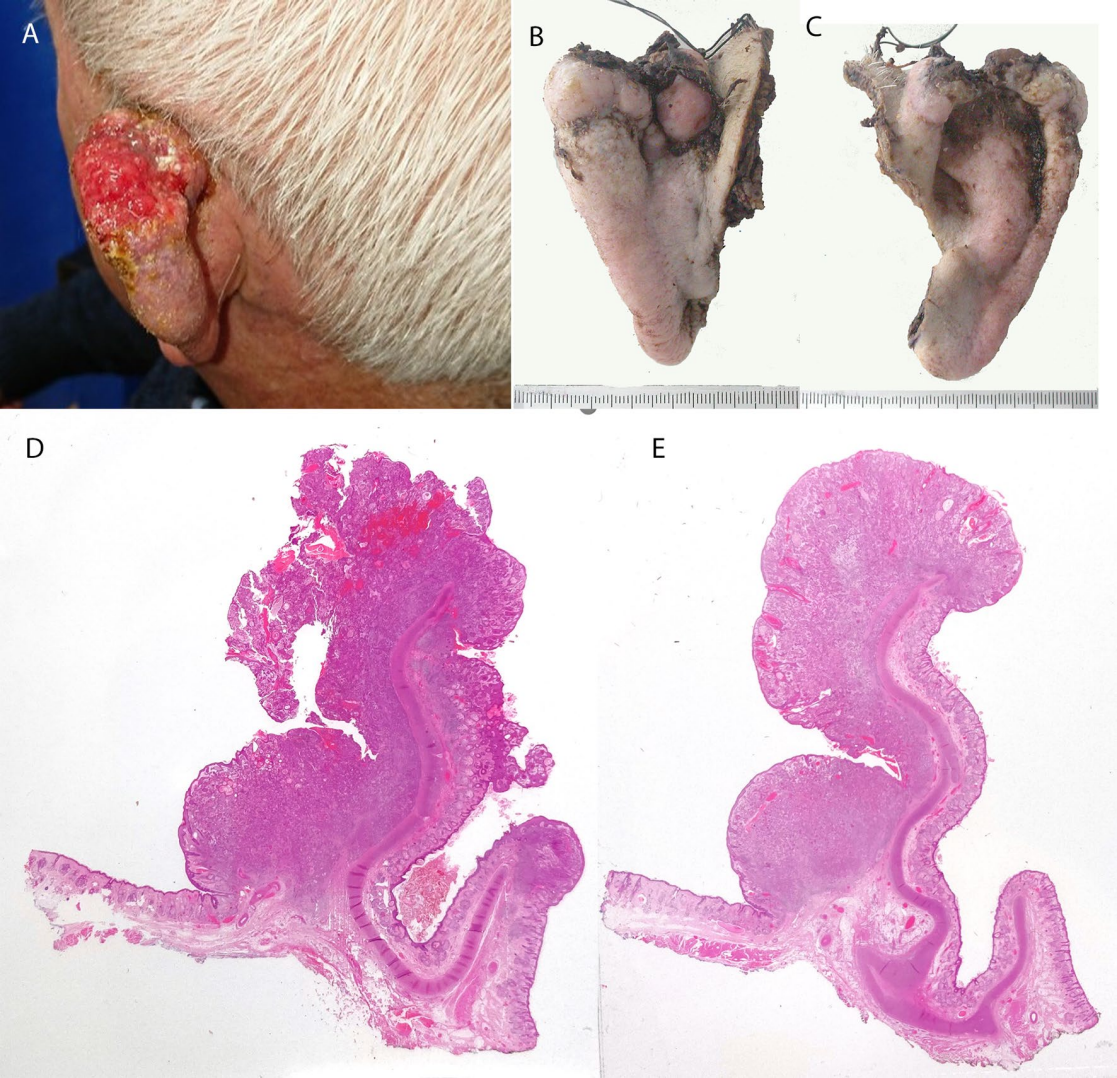
Squamous cell carcinoma of pinna. **A** Clinical picture of advanced squamous cell carcinoma of pinna. **B** Anterior & **C** Posterior macroscopic images of the pinnectomy specimen. The tragus and ear canal has been preserved. **D**, **E** Wholemount H&E stained sections of axial slices through the resection specimen. The tumour is exophytic and completely excised

**Fig. 4:**
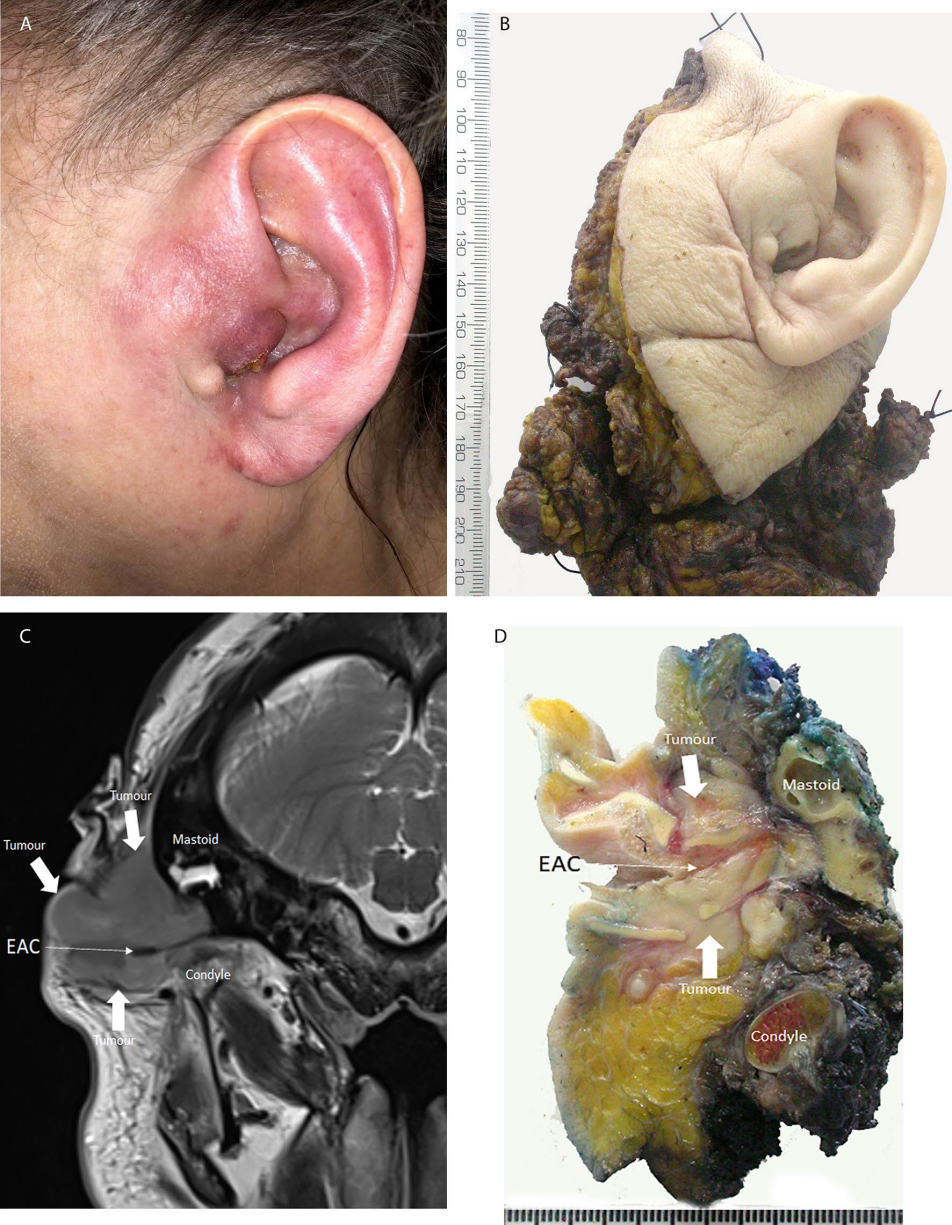
Squamous cell carcinoma of external ear canal. Patient presented late and treatment required total pinnectomy and resection of lateral temporal bone and temporomandibular joint. **A** Clinical image of SCC in EAC. **B** Macroscopic image of the resection specimen left total pinnectomy lateral temporal bone and temporomandibular joint. **C** Axial MRI image annotated to show the tumour and related anatomy*. **D** Macroscopic image of axial slice through the ear canal annotated like the radiological image to show tumour and related anatomy. *Image is courtesy of Dr Phil Touska Consultant Head Neck Radiologist Guys & St Thomas’s NHS Foundation Trust London UK

The histological appearance of squamous cell carcinoma of the external and middle ear is similar to that described at any other site in the body. Lesions affecting the skin of the pinna are often recognised early but the skin of the external ear canal (EAC) is hidden from view and access for examination and biopsy is limited. Surgery on the pinna is aimed at organ preservation and achieving best cosmetic result (Fig. 3). Problems arise in diagnosis of EAC lesions due to inadequacy of biopsies and lack of expertise in interpretation. Treatment is predominantly surgical; lesions often present late and extensive surgery may be required. Histological examination of the resection specimens may be problematic for the surgical pathologist who must interpret the dimensions of the tumour for staging and to assess the margins (Fig. 4). A recent update on examination and staging of squamous cell carcinoma of Ear and temporal bone [12] addresses the complex anatomy and the approach to macroscopic examination. The approach and techniques can of course be applied to tumours other than SCC occurring at this site that require surgical intervention.

## Tumours of Middle and Inner Ear

Tumours of the middle ear are rare. Due to the proximity to skull base, nasopharynx and paranasal sinuses the differential diagnosis is broad and representative biopsy difficult to obtain. Biopsies must be interpreted in the light of comprehensive clinical information and imaging reports wherever possible.

### Otosclerosis

Otosclerosis accounts for up to 9% of all hearing loss and is the commonest cause of conductive hearing loss [13]. It results from abnormal bone formation in the otic capsule causing plaques of softer, more vascular bone to replace the otic capsular endochondral bone. These plaques can spread to involve the stapes footplate impeding the action of the ossicle and causing conductive hearing loss. The resected stapes is the commonest biopsy sample but diagnostic lesional tissue is infrequently represented.

It is traditionally regarded as a middle ear disease, but it can affect inner ear causing sensorineural hearing loss, tinnitus and vertigo [14]. Therefore, patients can present with mixed (both conductive and sensorineural) hearing loss. Disease is usually bilateral (80%) and asymmetrical developing in one ear before the other is affected. Clinically the disease affects mainly Caucasians (prevalence 0.3–0.4%) [15] with a reported incidence in White Europeans of 0.1–2.1% [14] and is rare in Africans and Asians (0.03–0.1%). Incidence in Japanese and South American populations is half that in Caucasians [16]. Histological otosclerosis identified in a large autopsy series is 8–11%. Age at presentation is variable, typically 3rd decade (range 1st–6th decade) [17–19]. The disease is more often diagnosed in females reported ratio about 2:1 [14, 20, 21].

The histological characteristic of otosclerosis is the presence of trabeculae of spongy new woven bone with numerous enlarged plump osteoblasts and dilated richly vascular marrow spaces containing cellular connective tissue with many osteoclast giant cells [13]. These zones of active resorption (otospongiosis) contrast with the well-developed lamellar bone beneath outer periosteum, endochondral middle layer and the endosteal layer of the otic capsule. A sharply demarcated edge between normal and otosclerotic bone being a prominent feature. Otosclerotic bone sometimes reaches the endosteum of the cochlear capsule. In some cases, it may lead to a fibrous reaction deep to the spiral ligament. These changes are probably the basis of the sensorineural hearing loss that is also occasionally found in cases of otosclerosis.

Histologically active and inactive areas are described. Active areas (otospongiosis) are zones of active bone resorption composed of spongy immature bone with numerous enlarged plump osteoblasts and dilated richly vascular marrow spaces containing cellular connective tissue with many osteoclast giant cells [13].

The disease is regarded as a disorder of bone remodelling and linked to COL1A1 gene expression. Different autoantibodies, inflammatory cytokines, and growth factors have been implicated. Measles virus has been detected in osteosclerotic plaques, but the role remains controversial. It may trigger inflammatory processes in the active phase of disease. Oestrogen deficiency is thought to cause otosclerosis in menopausal women and hormone replacement has been shown to improve symptoms [22]. The mode of spread of the ostosclerotic plaques into normal tissue and the growth and persistence of the disease throughout life as evidenced by autopsy studies suggests that otosclerosis represents a low-grade neoplasm [23].

### Cholesteatoma

Cholesteatoma results from the abnormal proliferation of keratinising epithelium in the middle ear space beneath the tympanic membrane. The benign histology belies the aggressive and destructive nature of the disease [24]. The clinical staging of cholesteatoma is included in the 5th edition to correlate with extent of disease and difficulty with surgical resections and preservation of hearing.

**Fig. 5:**
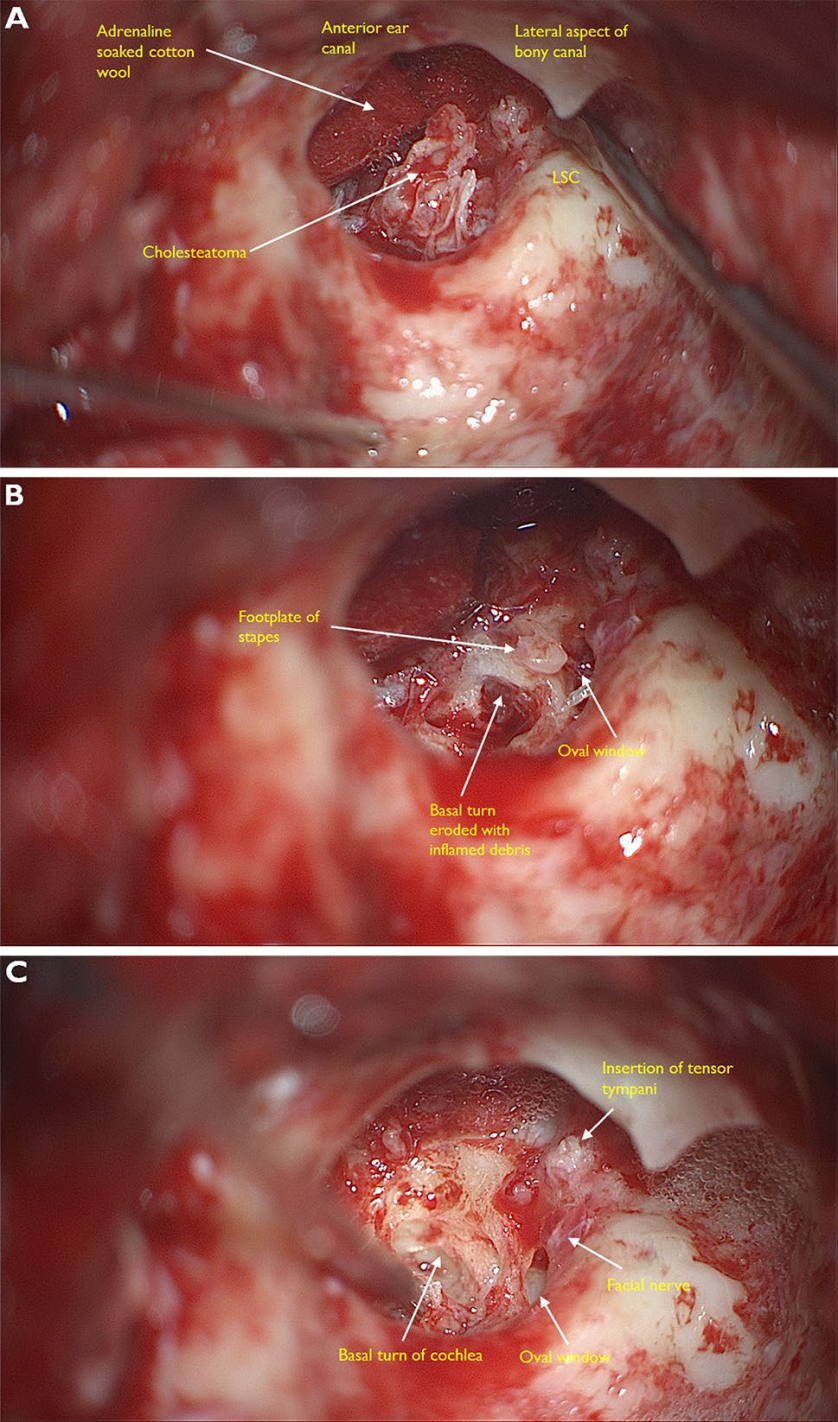
Intraoperative microscopic views taken at mastoidectomy for cholesteatoma*. **A** The ear canal has been taken down and we can see into the middle ear. The cholesteatoma appears as a friable white mass. **B** This image shows erosion of the footplate of the stapes. **C** On further dissection the anatomy of the cochlear is clearly visible. Round window is totally eroded and there is complete destruction of the cochlea promontory. The tympanic segment of the facial nerve is usually in a bony canal which can sometimes be dehiscent (~ 10% of patients with healthy ears). Here it is exposed, possibly inflamed and damaged due to irritation from the overlying cholesteatoma. *Images courtesy Mr Harry Powell Consultant ENT surgeon & Dr Charlotte Arnold (trainee ENT surgeon) Guys & St Thomas’s NHS Foundation trust London UK

There are varying opinions concerning the origin of cholesteatoma. Primary or congenital cholesteatoma is thought to arise from embryological rests of squamous epithelium in the middle ear, the so called ‘epidermoid formation’ from which keratin filled cysts develop behind the intact tympanic membrane [25]. Otitis media may result in chronic inflammation of the tympanic membrane resulting in a retraction pocket or ingrowth or downward migration of keratinising squamous epithelium into the middle ear and subsequent development of cholesteatoma. The cholesteatoma appears as a white mass or ‘wallpaper’ within the middle ear and can infiltrate and destroy ossicular bone (Fig. 5). Biopsies usually show only cytologically bland keratinising squamous epithelium and abundant keratin flakes.

### Middle Ear Papilloma

Sinonasal type middle ear papillomata are included in this chapter, named temporal bone sinonasal type papillomata (TBSP) that show predominantly inverted architecture and morphology with occasional oncocytic subtypes described. The lesions usually present as a unilateral middle ear mass but commonly concurrently affect the external ear canal as well as mastoid, eustachian tube and skull base. In about half of cases the sinonasal tract and nasopharynx are concurrently involved by disease [26–32]. Association with low-risk HPV has been reported [33–35] and in rare cases transcriptionally active high-risk HPV DNA has been detected [35, 36]. No EGFR or KRAS mutations are described [37, 38]. The tumours are aggressive with high risk of recurrence and 10% mortality [32].

Two distinct papillary cystic tumours have recently been described at this site one resembling an oncocytic sinonasal papilloma and another resembling salivary intraductal papillary mucinous neoplasm (IPMN). The patients did not have Von Hippel Lindau disease. The tumours were tested for mutations and gene fusions known to be associated with sinonasal and salivary neoplasms with similar morphology. The sinonasal papilloma—like tumour was found to harbour a fusion gene *MKRN1-BRAF* that has not been identified in sinonasal papillomata or ELST. No gene fusions were detected in over 500 genes tested in the salivary type tumours [39]. This suggests there is a spectrum of papillary cystic tumours affecting the middle ear, distinct from ELST and the relationship between the subtypes remains to be clarified.

### Vestibular Schwannoma

Vestibular schwannoma is the commonest temporal bone neoplasm and accounts for most cerebellopontine angle tumours. Unilateral sporadic tumours account for 5–10% of intracranial tumours. They arise most commonly at the glial-neurilemmal junction of the eighth nerve, which is usually within the internal auditory meatus. There is a causal relationship between mutations in the NF2 gene resulting in loss of expression of MERLIN or schwannomin and tumour development [40]. Severity of disease and mortality appears to be related to the type of mutation [41]. Tumours associated with neurofibromatosis type 2 (NF2) are often bilateral and occur at a younger age. Inactivating mutations in the NF2 gene have been described in up to 75% of sporadic tumours [42–45].

Vestibular schwannoma is indistinguishable histologically from schwannoma occurring at other sites. The NF2 associated tumours are similar to sporadic tumours but are described as having more Verocay bodies and more foci of high cellularity. The NF2 tumours tend to be more invasive and infiltrate the cochlea and vestibule more deeply.

As with all schwannomas, vestibular schwannoma, whether sporadic or NF2 associated, shows diffuse strong expression of S100 protein and SOX 10 [46, 47] and vimentin is also usually positive. An increased proliferation index has been associated with more aggressive clinical behaviour and metastasis.

### Middle Ear Neuroendocrine Tumour (MeNET)

Middle ear adenoma has been renamed middle ear neuroendocrine tumour (MeNET) according to the nomenclature of neuroendocrine tumours at other sites. Prior to the 4th edition of the WHO Classification of Head and Neck Tumours a name with a similar abbreviation was proposed for these tumours to reflect the apparent exocrine and neuroendocrine differentiation and the unpredictable clinical behaviour with the risk of local recurrence and metastasis. This name ‘mixed epithelial neuroendocrine tumour’ (MENET) was then considered a more accurate moniker [48].

More recent studies suggest the tumours contain a single cell population of cuboidal to columnar cells rather than a dual cell population including myoepithelial cells [5, 49]. The neoplastic cells are positive for high and low molecular weight keratins (AE1/AE3, Cam5.2) and neuroendocrine markers (synaptophysin, chromogranin) as well as transcription factors INSM1, islet-1 (ISL1) and SATB2. Immunostains for CDX2 and GATA3 may be focally expressed but TTF1 is negative which may be diagnostically useful to exclude lung metastasis. The tumours produce hormones, most commonly glucagon, pancreatic polypeptide, and PYY, but serotonin is also expressed [49–52].

These infiltrative architecturally variable tumours mostly show a very low proliferation rate but rates up to 20% have been reported. Increased proliferation rate has been associated with local recurrence and metastasis. Grading based on tumour proliferation rate on ki67 immunostaining is being investigated in line with the G1 G2 G3 grading generally in use for other neuroendocrine tumours [49, 53].

### Middle Ear Squamous Cell Carcinoma

The most common malignancy of middle ear is conventional keratinising squamous cell carcinoma. Non keratinising squamous cell carcinoma primary in middle ear is extremely rare. Sinonasal non keratinising carcinomas have been associated with HPV and EBV virus infection which may help to exclude secondary malignancy. Recently 2 cases of primary non keratinising SCC of middle ear have been described which were found to be associated with DEK-AFF2 fusion gene [54]. This is clinically relevant because case reports in the literature have described tumours at other sites associated with this gene fusion have responded well to programmed death—ligand 1 (PDL1) inhibitor therapy. Since surgery of temporal bone is difficult and morbid alternative treatment in terms of immunotherapy is an important avenue to explore.

### Endolymphatic Sac Tumour & Middle Ear Adenocarcinoma

Papillary cystic tumours of the middle ear included in the 4th edition of the WHO Classification of Head and Neck Tumours included only endolymphatic sac tumours and aggressive papillary tumour of middle ear. In the 5th addition the term ‘aggressive papillary tumour’ has been dropped in favour of ‘middle ear adenocarcinoma’. Von Hipple Lindau mutation has been described in association with papillary tumours involving middle ear that may have arisen in endolymphatic sac. Tumours confined to middle ear without involvement of endolymphatic sac and negative for VHL mutation likely represent a subset of adenocarcinoma distinct from ELS tumour [55, 56]. Analogous papillary tumours of middle ear have developed in a mouse model in which EGFR was mutated [57].

Most tumours show a papillary glandular pattern with complex architecture including papillae lying loosely or infiltrating fibrous connective tissue. The papillae are lined usually by a single layer of low cuboidal to columnar epithelial cells with uniform nuclei, eosinophilic cytoplasm and indistinct cell borders. Thyroid follicle-like areas may be present similar to those seen in endolymphatic sac tumour.

Immunostains for CK7, EMA are positive, and they may express S100. The main differential is ELST which stains positively for PAX8 and CAIX [58] while adenocarcinoma primary in middle ear is negative. Metastasis from tumours in lung, thyroid and colon can be excluded by immunostains for thyroglobulin, TTF1, CK20 and CEA.

Primary tumours of the inner ear are very rare. Case reports of tumours in the inner ear refer to tumours at the cerebellar pontine angle or internal acoustic meatus. A study of 426 lesions affecting the cerebellar pontine angle reported from New York USA in 1997 showed just over 90% were schwannoma (acoustic neuroma) and 4% were meningiomas [59]. On imaging lesions affecting the inner ear or cerebellar pontine angle are most often diagnosed as vestibular schwannoma and the differentials will be benign including meningioma, arachnoid cyst, haemangioma or lipoma. The inner ear may be affected by direct extension of malignant tumours in external or middle ear, brain, nasopharynx or base of skull. Distant metastasis to the temporal bone has been described. A recent international multicentre study included a series of 9 cases of secondary malignancy affecting the cerebellar pontine angle/internal acoustic meatus [60]. There were 3 cases of metastatic breast carcinoma, 2 lung carcinoma metastases, 2 B-cell lymphomas, a Grade II atypical meningioma and adenocarcinoma metastatic from ethmoid. Most of the patients presented with profound hearing loss and facial nerve palsy. Diagnosis was made only after surgery in five patients, after lumbar puncture in three patients and on PET imaging in 1 patient.

Endolymphatic sac tumour (ELST) is known to be associated with Von Hippel Lindau (VHL) disease and sporadic tumours also show mutations in the VHL gene on the short arm of chromosome 3 (3p26.3). Data from the International ELST registry showed a prevalence of 3.6% of ELST in VHL syndrome. Sporadic tumours were shown to carry germline mutation in the VHL gene in 39% of cases and 32% of VHL patients initially presented with ELST. Therefore, patients with ELST should be screened for VHL and patients with VHL should be screened for ELST [61–65]. Tumours in patients with VHL tend to be smaller but the lesions may be bilateral in about 30%. ELST is a low grade locally aggressive lesion that arises in a characteristic location in the vestibular aqueduct. Larger lesions expand to involve the temporal bone and middle and posterior cranial fossae. Lesions greater than 30 mm may cause facial nerve palsy. Symptoms from smaller lesions are nonspecific. Patients may present with a feeling of ‘fullness’ in the ear, loss of balance or sensorineural hearing loss. ELST usually has a papillary-glandular appearance with vascular papillae lined by a single row of low cuboidal cells. There may be areas of dilated glands containing colloid like secretion which may dominate the histological pattern. A few cases are mostly composed of clear cells which may mimic resembling metastasis from kidney.

The neoplastic cells are positive for pan cytokeratin, CK7, HIF-1α, EMA, GLUT1, CAIX (membranous) and PAX-8 (nuclear). There is variable expression of S100 protein, GFAP, and vimentin. There is no expression of TTF-1, thyroglobulin, PSA, CD10, P504S, p63, synaptophysin, GATA3, and RCC [58, 66–72]. The proliferation rate with Ki67 immunostain is low.

Treatment is based on relieving audio vestibular symptoms and surgery on visible tumours to preserve hearing. Radiotherapy has been used to treat large or unresectable tumours, but numbers of cases are too few to assess efficacy [73].

A recent Italian study reporting 13 cases and review of the literature concluded that ELST may go undetected for long periods particularly in sporadic cases and the rarity causes difficulty in diagnosis both clinically and histologically. In larger lesions the characteristic site of origin may be obscured by extension to involve adjacent structures. Recurrence was associated with under-estimation of the extent of temporal bone invasion by the tumour. Eradication may involve extensive surgery with excision of inner ear structure causing loss of hearing [74].

## Conclusion

The approach to the diagnosis and management of lesions in the ear remains unchanged—careful orientation and communication with clinical surgeon and radiologist colleagues is essential and this is emphasised in the 5th edition chapter. Biopsies from the ear are difficult to obtain and often not representative. Inexperienced pathologists should consider referring biopsies, particularly from the external ear, representing lesions that appear atypical, or that are recurrent, for review by a specialist multidisciplinary team.

The use of standardised nomenclature and evidence based minimum data sets for resection specimens will help deliver optimal management and maximise the data available for the investigation of origin, aetiology and pathogenesis of the rare lesions occurring in ear and temporal bone.
